# Nitrite Triggers Reprogramming of the Oral Polymicrobial Metabolome by a Commensal Streptococcus

**DOI:** 10.3389/fcimb.2022.833339

**Published:** 2022-03-01

**Authors:** Joshua T. Huffines, Sara N. Stoner, Joshua J. Baty, Jessica A. Scoffield

**Affiliations:** Department of Microbiology, University of Alabama at Birmingham, Birmingham, AL, United States

**Keywords:** oral commensal, nitrite, polymicrobial, bacterial competition, reactive nitrogen species

## Abstract

Commensal streptococci regulate health and homeostasis within oral polymicrobial communities. Remarkably, high salivary nitrite concentrations have also been associated with improved health in the oral cavity. We previously demonstrated that nitrite assists hydrogen peroxide-producing oral commensal streptococci in regulating homeostasis *via* the generation of reactive nitrogen species (RNS), which have antimicrobial activity on oral pathogens. However, it is unknown how nitrite and commensal streptococci work in concert to influence the metabolome of oral polymicrobial communities. In this study, we report that nitrite aids commensal streptococci in the inhibition of multi-kingdom pathogens that reside in distinct oral niches, which supports commensal dominance. More importantly, we show that commensal streptococci utilize nitrite to drive the metabolic signature of multispecies biofilms in a manner that supports commensal metabolism and resistance to RNS, and restricts metabolic processes that are required for pathogen virulence. Taken together, our study provides insight into how commensal streptococci use nitrite to trigger shifts in the oral polymicrobial metabolome to support health and homeostasis.

## Introduction

There are approximately 700 resident microbes in the oral cavity (Dewhirst et al., 2010). These microbes include cariogenic, endodontic, and periodontal pathogens, but also commensals whose primary function is to maintain health and homeostasis (Dewhirst et al., 2010; Agnello et al., 2017; Bryan et al., 2017). As primary colonizers of the oral cavity, commensal streptococci influence the structure of dental plaque communities by antagonizing pathogens *via* the production of hydrogen peroxide (H_2_O_2_) (Jakubovics et al., 2008; Kreth et al., 2008; Zhu and Kreth, 2012). Commensal-generated H_2_O_2_ has been shown to inhibit the periodontal pathogen *Aggregatibacter actinomycetemcomitans*, and also mediate cell signaling in multispecies biofilms that result in the promotion of commensal biofilm development (Duan et al., 2016). Further, a number of studies have demonstrated that H_2_O_2_-producing streptococci, including *Streptococcus parasanguinis*, inhibit the cariogenic pathogen *Streptococcus mutans* (Jakubovics et al., 2008; Kreth et al., 2008). Altogether, these studies illustrate that H_2_O_2_ is a primary mediator of bacterial competition and homeostasis in the oral cavity.

*Streptococcus mutans* is a main etiological agent of dental caries (Bowen and Koo, 2011). *S. mutans* can persist within and dominate biofilms in the oral cavity by metabolizing dietary carbohydrates into lactic acid, tolerating acid stress, and producing insoluble glucan, a sucrose-dependent exopolysaccharide (EPS) that is synthesized by glucosyltransferases (GTFs) (Ahn et al., 2008; Koo et al., 2013). Further, the fungal pathobiont *Candida albicans* is frequently co-isolated with *S. mutans* from carious lesions in individuals with early childhood caries (Falsetta et al., 2014; Hwang et al., 2017), which is one of the most prevalent infectious diseases worldwide. Together, *S. mutans* and *C. albicans* interact synergistically, leading to increased biofilm biomass and more severe tooth decay compared to a *S. mutans* single infection in a rat model of dental caries (Hwang et al., 2017). Further, exogenous GTFs synthesized by *S. mutans* promote *C. albicans* growth by breaking down sucrose into fructose and glucose, which can be readily metabolized by *C. albicans*, and GTFs facilitate incorporation of *C. albicans* into oral biofilms (Gregoire et al., 2011; Falsetta et al., 2014; Hwang et al., 2017). Although the cross-kingdom synergy displayed between *S. mutans* and *C. albicans* is one of the most notable and well-studied polymicrobial interactions in the oral cavity, few studies have examined how oral commensal bacteria modulate the interaction between these microbes.

While commensal generated H_2_O_2_ has been regarded at the major antagonist of oral pathogens, other commensals such as *Prevotella* and *Veillonella* can generate bactericidal nitrogenous intermediates through the process of denitrification by reducing nitrate to nitrite, and nitrite to nitric oxide (NO) (Hyde et al., 2014). Interesting, nitrate and nitrite have been implicated in regulating oral health. High salivary nitrite and nitrate levels (> 1mM) and high nitrate-reducing capability by oral commensal bacteria have been associated with a reduced incidence of dental caries (Doel et al., 2004). Additionally, consuming foods rich in nitrate has been shown to induce anti-cariogenic effects by increasing salivary nitrite levels and, as a result, increase salivary pH (Hohensinn et al., 2016). Moreover, we have previously reported that H_2_O_2_ produced by the commensal *S. parasanguinis* can react with dietary or exogenous nitrite to produce reactive nitrogen species (RNS) that have antimicrobial activity against *S. mutans* and the respiratory pathogen *Pseudomonas aeruginosa* (Scoffield and Wu, 2015; Scoffield and Wu, 2016; Scoffield et al., 2019). The observation that commensal generated H_2_O_2_ and nitrogenous intermediates work in concert to modulate oral health is a new concept, however, how these metabolites regulate the structure and function of the oral polymicrobial biofilm are largely unknown.

The importance of studying how metabolic exchange influences polymicrobial communities and regulates the fitness of cohabitating organisms to affect health outcomes is becoming increasingly recognized. A multi-omics study demonstrated that in the presence of *S. mutans*, *C. albicans* genes that drive carbohydrate metabolism were significantly upregulated (Ellepola et al., 2019), which presumably can promote caries development. We previously demonstrated the ability of *S. parasanguinis* to inhibit the growth of *S. mutans* and caries development in a H_2_O_2_- and nitrite-dependent manner using a rat caries model (Scoffield et al., 2019). Further, we have demonstrated that *S. parasanguinis* disrupts synergy between *S. mutans* and *C. albicans* in a contact and H_2_O_2_-independent manner (Huffines and Scoffield, 2020). While bacterial and host-derived metabolites are known to shape oral polymicrobial communities and nitrite has been shown to aid oral commensal streptococci in the inhibition of diverse pathogens, no studies have observed how oral commensals such as *S. parasanguinis* influence the metabolomes of polymicrobial communities in the presence of nitrite.

In this study, we tested the role of nitrite on the oral polymicrobial metabolome by utilizing a three-species biofilm model containing *S. mutans*, *C. albicans*, and *S. parasanguinis*. We demonstrate that *S. parasanguinis* biofilm development is promoted in complex polymicrobial biofilms in the presence of nitrite. Further, metabolomics studies revealed that both *S. parasanguinis* and nitrite alter the polymicrobial metabolic signature of dual and triple species biofilms. Additionally, *S. parasanguinis* displays a growth advantage in the presence of nitrite, while *S. mutans* and *C. albicans* are nitrite-sensitive. Moreover, *S. parasanguinis* upregulates production of RNS scavengers, which potentially explains why *S. parasanguinis* is capable of tolerating high nitrite concentrations. Finally, thioproline, a nitrite trapping antioxidant that is a product of *S. parasanguinis* metabolism, is inhibitory to glucan production, a virulence factor produced by *S. mutans*. Taken together, our results support a model in which oral commensal streptococci use nitrite to drive the metabolic signature of polymicrobial biofilms in a manner that supports commensal metabolism and protects against RNS, as well as restricts pathogen virulence factor production, thus promoting health and homeostasis.

## Materials and Methods

### Bacterial Strains and Growth Conditions

*S. parasanguinis* FW213, *S. mutans* UA159, and *C. albicans* SC5314 were used in this study. *S. parasanguinis* and *S. mutans* were grown in Tryptic Soy Broth with 5% yeast extract (TSBYE). *C. albicans* was maintained on Todd Hewitt agar and grown in yeast peptone dextrose (YPD). For polymicrobial experiments TSBYE was used at all times. All cultures were grown at 37°C with 5% CO_2_. For growth curves, strains were grown in TSBYE (+/- 2mM NO_2_ and/or 2 mM thioproline) in a 96 well plate and incubated and read in a BioTek Synergy H1 plate reader.

### Biofilm Formation and Confocal Laser Scanning Microscopy (CLSM)

Overnight cultures of *S. parasanguinis* (mCherry)*, S. mutans* (GFP), or *C. albicans* were subcultured and grown to an optical density of 0.5 at an absorbance of 600nm. All biofilms were grown in TSBYE + 1% sucrose with or without 2mM nitrite in µ-Slide 8 well slides (Ibidi, Gräfelfing, Germany, Cat #: 80826) and all inocula were seeded at 1x10^4^ CFU/mL. Biofilms were allowed to grow for 16 hours at 37°C with 5% CO_2_. All biofilm wells were washed with PBS and wells with *C. albicans* were stained with calcofluor white for 15 minutes before imaging. A Nikon A1 + confocal laser scanning microscope (CLSM) (Nikon Instruments Inc., Melville, NY, USA) was used to image biofilms at 60x magnification and 3D images were acquired using the Nis Elements 5.0 Imaging Software (Nikon Instruments Inc., Melville, NY, USA). To enumerate colony forming units, all biofilms were gently washed with sterile PBS twice before adding 200 µL of sterile PBS for plating. The biofilms were scraped up with a 200 µL tip, vortexed for 10 s, and serially diluted. All dilutions were plated on Todd-Hewitt Broth or blood agar plates and incubated at 37 °C with 5% CO_2_ for a minimum of 16 h before counting.

### Metabolomics Analysis

Five biological replicates of single, dual, and three species biofilm cultures were grown overnight in 20mL of TSBYE+1% sucrose (+/- 2 mM NO_2_) in 50mL conical tubes to ensure that we could obtain 100 µL of packed cells as required by Metabolon, Inc (Durham, NC, USA). Samples were harvested and stored at -80°C prior to shipment on dry ice to Metabolon, Inc. To analyze metabolites, samples were prepared using the automated MicroLab STAR^®^ system from Hamilton Company. To remove protein, dissociate small molecules bound to protein or trapped in the precipitated protein matrix, and to recover chemically diverse metabolites, proteins were precipitated with methanol under vigorous shaking for 2 min (Glen Mills GenoGrinder 2000) followed by centrifugation. The resulting extract was divided into five fractions: two for analysis by two separate reverse phase (RP)/UPLC-MS/MS methods with positive ion mode electrospray ionization (ESI), one for analysis by RP/UPLC-MS/MS with negative ion mode ESI, one for analysis by HILIC/UPLC-MS/MS with negative ion mode ESI, and one sample was reserved for backup. Samples were placed briefly on a TurboVap^®^ (Zymark) to remove the organic solvent. Peaks were quantified using area-under-the-curve. All samples were analyzed for raw counts using liquid chromatography-mass spectrometry (LCMS). Raw counts were median scaled with missing values imputed with the lowest value.

### GTF Precipitation, Cell-Free Glucan Formation, and Glucan Quantification

Overnight cultures of *S. mutans* supernatants were ethanol precipitated at a 1:1 ratio of 100% ethanol to precipitate extracellular GTFs. Supernatants were incubated at −80°C for 1 h, pelleted, and re-suspended in fresh TYE ( ± 1% sucrose). To test the role of nitrite and thioproline on glucan formation, 100 µL of cell-free GTFs were added to 1 mL of TSBYE media that contained no sucrose, 1% sucrose, or 1% sucrose with 2mM nitrite, 2mM thioproline, or both. For all experiments, 1 µM dextran-conjugated Cascade Blue (Molecular Probes, Invitrogen) was added to the media before overnight incubation. Samples were dispensed in Ibidi slides and incubated for 16 h at 37°C+5% CO_2_ to permit glucan formation. Fluorescence was quantified using ImageJ.

### Statistical Analysis

For colony-forming units and glucan formation assays, we analyzed the data using Prism version 8.4.3 (GraphPad Software, LLC). A *P*-value of 0.05 was used to determine statistical significance. A minimum of three biological replicates (with 3 technical replicates for all experiments except glucan quantification, which contained 2 technical replicates per experiment) were completed for each experiment. The metabolomics data consisted of 5 replicates and one-way ANOVA was used to determine *P* values for the single-species group and a two-way ANOVA was used for groups with multiple species. Metabolomics data was assessed using principal components analysis (PCA) in R. Heatmaps were created using GraphPad Prism.

## Results

### Nitrite Shifts the Oral Polymicrobial Biofilm Toward Commensal Dominance

**Figure 1:**
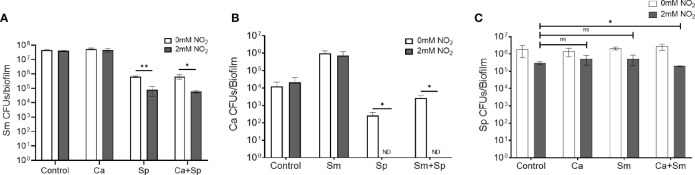
*S. parasanguinis* reduces *S. mutans* and *C. albicans* biofilm formation in a nitrite-dependent manner. Colony forming units of 16-hour single-, dual-, and tri-species biofilms with or without 2mM NO_2_. **(A)** *S. mutans* (Sm), **(B)** *C*. *albicans* (Ca), and **(C)** *S. parasanguinis* (Sp). All biofilms were grown for 16 hours in tryptic soy broth containing 0.5% yeast extract and 1% sucrose. *p < 0.05, **p < .001, and ns, not significant. ND, not detected. (Student’s t-test) Data are representative of 3 biological replicates that contained 3 technical replicates.

Previous studies have demonstrated that nitrate and nitrite derivatives and commensal streptococci play a prominent role in maintaining oral health, presumably through the generation of RNS that display antimicrobial activity (Hyde et al., 2014; Hezel and Weitzberg, 2015; Qu et al., 2016). *S. mutans* and *C. albicans* have been shown to engage in metabolic crosstalk that promotes carious lesions in individuals with severe early childhood caries (Hwang et al., 2017). However, no studies have examined how commensal and nitrite-mediated activity alter the oral polymicrobial metabolome and interfere with the metabolic cross-talk of *S. mutans* and *C. albicans*, which is the most notable example of microbial metabolic synergy in the oral cavity. Using a complex three species biofilm model between an oral commensal (*S. parasanguinis*), a cariogenic pathogen (*S. mutans*), and a fungal pathobiont (*C. albicans*), we first probed the impact of *S. parasanguinis* and nitrite-mediated activity on the oral polymicrobial biofilm. Consistent with previous findings, *S. parasanguinis* inhibited *S. mutans* in a nitrite dependent manner in the dual species biofilm, but also in the triple species biofilm with *C. albicans* (**Figure 1A**). Interestingly, *S. parasanguinis* completely inhibited *C. albicans* growth in both dual and triple species biofilms (**Figure 1B**). Nitrite had no significant impact on single or dual species biofilms containing *S. mutans* and *C. albicans* (**Figures 1A, B**). Remarkably, the abundance of *S. parasanguinis* did not dramatically change in dual or triple species cultures compared to single species cultures with nitrite (**Figure 1C**). Additionally, the activity of *S. parasanguinis* and nitrite had broad antimicrobial activity on oral pathogens. *S. parasanguinis* and nitrite inhibited a clinical isolate of *Enterococcus faecalis* that was isolated from an endodontic infection, as well as the periodontal pathogen *A. actinomycetemcomitans* (**Figure S1**). Taken together, these results demonstrate that *S. parasanguinis* and nitrite display broad antimicrobial activity against diverse oral pathogens, either within a single culture or a complex polymicrobial biofilm.

### *S. parasanguinis* and Nitrite Reprogram the Metabolic Signature of Oral Polymicrobial Biofilms

**Figure 2:**
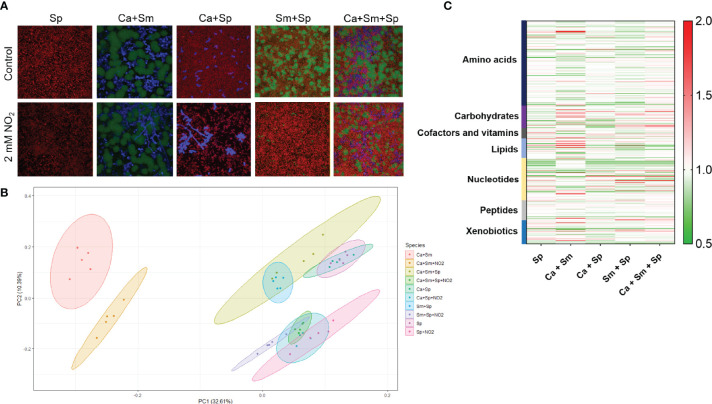
*S. parasanguinis* and nitrite reprogram the metabolic signature of oral polymicrobial biofilms **(A)**. Confocal scanning laser microscopy images of *S. parasanguinis* (Sp), *S. mutans* (Sm), *C*. *albicans* (Ca) in mixed biofilms at 60X magnification. *S. mutans* was labeled with green fluorescent protein (GFP), *C. albicans* was stained with calcofluor white, and *S. parasanguinis* was labeled with mCherry. All biofilms were grown in tryptic soy broth containing 0.5% yeast extract (TSBYE) and 1% sucrose (+/- 2mM NO_2_) for 16 h at 37°C with 5% CO_2_. **(B)** Principal Component Analysis of single (Sp only) and dual and triple species biofilms. **(C)** Global metabolomics profiling of dual- and tri-species cultures and single-species *S. parasanguinis* cultures. Metabolomics data were collected for 5 replicates in each group.

Due to the broad antimicrobial activity of *S. parasanguinis* and nitrite on oral pathogens, we utilized the prominent cross-kingdom synergistic model of *S. mutans*-*C. albicans* to probe how *S. parasanguinis* and nitrite drive the metabolic profiles of complex oral biofilm communities. First, we visualized the impact of *S. parasanguinis* and nitrite on the biofilm community structure of dual and triple species biofilms containing *S. mutans* and *C. albicans* using confocal microscopy. Similar to the results reported in **Figure 1**, the incorporation of *S. parasanguinis* reduced the presence of *S. mutans* in dual and triple species biofilms, and promoted the abundance of *S. parasanguinis* (**Figure 2A**). Although *C. albicans* could be visually detected in multispecies biofilms with *S. parasanguinis* and nitrite, the majority of the cells were not viable as indicated by a failure to recover colony forming units (**Figure 1**). Interestingly, the addition of nitrite in dual *S. mutans* and *S. parasanguinis* biofilms triggered the formation of hyphae by *C. albicans* (**Figure 2A**).

Given that the observed inhibitory phenotypes were largely driven by nitrite and *S. parasanguinis*, we analyzed the impact of nitrite on the *S. parasanguinis* metabolome, and also the effect of *S. parasanguinis* and nitrite on dual and triple species biofilms containing *C. albicans* and *S. mutans*. Principal component analysis revealed that nitrite facilitated a metabolic shift in *C. albicans* and *S. mutans* biofilms compared to when no nitrite was present (**Figure 2B**). When *S. parasanguinis* was added to *C. albicans* or *S. mutans* in dual biofilms, or in triple species biofilms with *C. albicans* and *S. mutans* containing nitrite, *S. parasanguinis* shifted the metabolomes of all polymicrobial cultures (**Figure 2B**). Generally, we observed major shifts in major classes of metabolites, including amino acids, nucleotides, lipids, and xenobiotics (**Figure 2C**). Overall, these results demonstrate that nitrite governs the dominance of *S. parasanguinis* in oral polymicrobial biofilms.

### *S. parasanguinis* Displays Increased Resistance to the Nitrite Trapping Antioxidant Thioproline Compared to *S. mutans*

**Figure 3:**
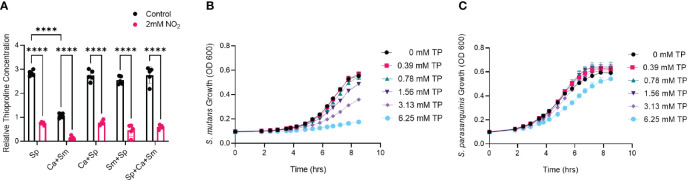
*S. parasanguinis* is resistant while *S. mutans* is sensitive to the nitrite trapping antioxidant thioproline. **(A)** Relative thioproline abundance from metabolomics analysis. **(B)** Growth of *S. mutans* on thioproline **(C)** Growth of *S. parasanguinis* on thioproline. All growth curve cultures were grown in tryptic soy broth containing 0.5% yeast extract (+/- 2 mM thioproline) at 37°C with 5% CO_2_. ****p < 0.0001. (Student’s t-test). Data are representative of three biological replicates.

A particular noteworthy observation from the metabolomics analysis revealed that single and polymicrobial cultures containing *S. parasanguinis* had elevated levels of thioproline with or without nitrite, whereas thioproline was produced at smaller concentrations in the *C. albicans* and *S. mutans* dual biofilm (**Figure 3A**). Thioproline is an antioxidant and analog of proline that has been shown to have nitrite trapping capability (Ham et al., 2020). Higher levels of thioproline in *S. parasanguinis*-containing cultures, but not *S. mutans* cultures prompted us to question whether the commensal may be more tolerant to thioproline compared to *S. mutans* since it produces this metabolite at a significantly higher concentration. To test whether thioproline supports commensal growth, we compared the growth of *S. parasanguinis* and *S. mutans* on various concentrations of thioproline. *S. mutans* was sensitive to every concentration of thioproline with the exception of 0.39 mM thioproline (**Figure 3B**). Surprisingly, *S. parasanguinis* was highly resistant, and even had a growth advantage on every concentration of thioproline compared to cultures with no thioproline, with the exception of very high levels (6.25 mM) of thioproline (**Figure 3C**). These data demonstrate that *S. parasanguinis* not only potentially uses thioproline to detoxify nitrite, but its increased tolerance to thioproline gives it a growth advantage over *S. mutans*, which could play a role in shifting the oral polymicrobial community toward commensal dominance.

### Nitrite and Thioproline Inhibit *S. mutans* Glucosyltransferase Activity

**Figure 4:**
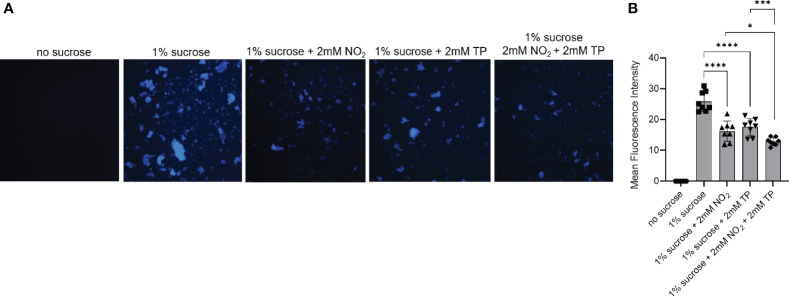
Nitrite and thioproline inhibit *S. mutans* glucosyltransferase activity. Fluorescence microscopy of purified GTFs from *S. mutans* cultures and cascade blue-labeled dextran in **(A)** TSBYE (no sucrose), TSBYE+1% sucrose, TSBYE+1% sucrose with 2mM NO_2_, 2mM thioproline (TP), or TSBYE+1% sucrose with 2mM NO_2_ and 2mM TP. **(B)** Fluorescence intensity of glucan production. *p < 0.05, ***p < 0.001, and ****p < 0.0001. (Student’s t-test). Data are representative of four biological replicates.

Our biofilm data demonstrated that *S. mutans* and *C. albicans* display decreased adherence in polymicrobial biofilms with *S. parasanguinis* in the presence of nitrite. Additionally, the growth of *S. mutans* is inhibited by the nitrite trapping metabolite thioproline. Altogether, these data suggest that nitrite directly interferes with the production of the major *S. mutans* biofilm matrix component glucan, and that thioproline is ineffective at protecting *S. mutans* against the effects of nitrite. *S. mutans* Gtf enzymes play a critical role in synthesizing glucan from sucrose (Bowen and Koo, 2011). Further, GtfB-mediated glucan production and binding to *C. albicans* mannan is considered to be a central mechanism of synergy between *C. albicans* and *S. mutans* (Hwang et al., 2017). Therefore, we reasoned that the inability of thioproline to protect against nitrite contributes to reduced adherence by *S. mutans* and *C. albicans* in polymicrobial biofilms with *S. parasanguinis* due to an interference in glucan production. To test this, we purified GTFs from cell-free *S. mutans* supernatant and cultured them in no sucrose or sucrose containing 2mM nitrite, 2mM thioproline, or both nitrite and thioproline. Using cascade blue to visualize the GTF-catalyzed glucan formation, we observed that nitrite inhibited glucan production and resulted in a decrease in fluorescence intensity compared to the sucrose only control (**Figures 4A, B**). Surprisingly, thioproline also decreased glucan production compared to the sucrose only control, and the combination of nitrite and thioproline further decreased the glucan and fluorescence intensity compared to either nitrite or thioproline (**Figures 4A, B**). Taken together, our results show that thioproline is not only inhibitory to *S. mutans* cell growth, but directly interferes with GTF activity and glucan production, and provides no protection against the effects of nitrite.

### *S. parasanguinis* Increases Nucleotide Metabolism and RNS Scavengers in Response to Nitrite

Our results illustrate that *S. parasanguinis* thrives in multispecies biofilms that contain nitrite. We have previously demonstrated that H_2_O_2_ produced by *S. parasanguinis* reacts with nitrite to produce RNS like peroxynitrite, which has antimicrobial activity on *S. mutans* and *P. aeruginosa* (Scoffield and Wu, 2015; Scoffield and Wu, 2016; Scoffield et al., 2019). Interestingly, *S. parasanguinis* displayed a growth advantage during exponential phase when grown on 2, 4 and 8 mM nitrite compared to cultures containing no nitrite (**Figure S2**), which suggests that this commensal is able to withstand elevated levels of nitrite-mediated nitrosative stress or RNS. However, *S. mutans* was sensitive to 4 and 8 mM nitrite and *C. albicans* was sensitive to both 2, 4 and 8 mM nitrite (**Figure S2**). Mechanisms of RNS stress resistance in *S. parasanguinis* are unknown, nevertheless, our findings reveal that *S. parasanguinis* is uniquely resistant to nitrite compared to other disease-causing oral microbes.

**Figure 5:**
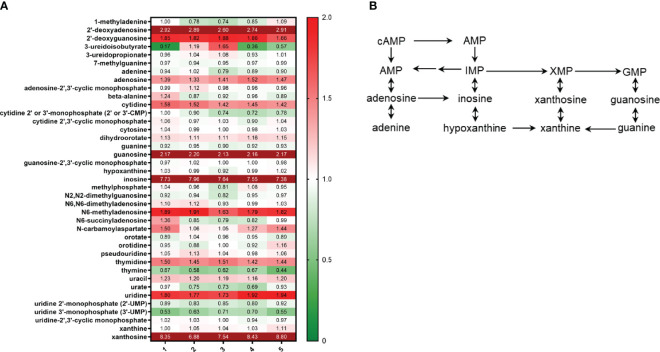
*S. parasanguinis* increases nucleotide metabolism in response to nitrite. **(A)** Heatmap of nucleoside metabolites (values are median normalized). **(B)** Nucleotide metabolic pathway. All cultures were grown for 16 hours in TSBYE and 1% sucrose in 5% CO_2_. Metabolomics data were collected for 5 replicates in each group.

**Figure 6:**
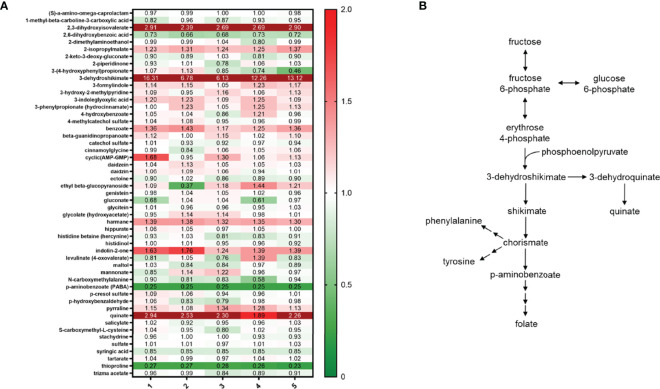
*S. parasanguinis* increases RNS scavengers and aromatic compounds in response to nitrite. **(A)** Heatmap of RNS scavengers and xenobiotic metabolites (values are median normalized). **(B)** Metabolic pathway for aromatic compounds. All cultures were grown for 16 hours in TSBYE and 1% sucrose in 5% CO_2_. Metabolomics data were collected for 5 replicates in each group.

As previously mentioned, metabolomics analysis demonstrated that *S. parasanguinis* cultures contained elevated levels of thioproline, a nitrite trapping antioxidant. Further, the ability of *S. parasanguinis* to resist high concentrations of nitrite suggests that this commensal bacterium is inherently more resistant to nitrosative stress than *S. mutans* and *C. albicans*, and has acquired mechanisms to cope with nitrosative stress. However, mechanisms used by *S. parasanguinis* to adapt to nitrosative stress are unknown. Our metabolomics data indicated that *S. parasanguinis* undergoes large shifts in nucleotide metabolism during growth on nitrite (**Figure 5A**). We observed increases in nucleoside turnover, particularly for xanthosine, inosine, and guanosine, which all feed into the nucleotide metabolic pathway (**Figure 5B**). We also detected large increases in aromatic compounds from the Shikimate pathway such as 3-dehydroshikimate and quinate that can be generated from fructose metabolism, in addition to the fatty acid 2 3-dihydroxy isovalerate (**Figures 6A, B**). Additionally, metabolites that are known to scavenge nitrite and ROS, such as p-aminobenzoate (PABA) (Hu M et al., 1995), were altered in *S. parasanguinis* cultures containing nitrite (**Figures 6A, B**), suggesting that *S. parasanguinis* can directly generate metabolites that detoxify RNS stress.

## Discussion

Bacterial competition plays a major role in health and disease where the host relies on commensal bacteria to out-compete pathogens by inhibiting microbial colonization and pathogenesis. In the oral cavity, one particular mechanism that mediates bacterial competition is the generation of RNS. We have previously published that the oral commensal *S. parasanguinis* produces H_2_O_2_ which can react with nitrite to form RNS, which has antimicrobial activity on a multitude of pathogens (Scoffield and Wu, 2015; Scoffield and Wu, 2016; Scoffield et al., 2019). H_2_O_2_ is a major mediator of pathogen antagonism in the oral cavity and its impact on maintaining oral health has been well documented (Regev-Yochay et al., 2006; Jakubovics et al., 2008; Kreth et al., 2008; Zhu and Kreth, 2012; Whiley et al., 2015). Conversely, the role of nitrite in the oral cavity is less understood. Nitrite is a metabolite that is derived from dietary nitrate through the denitrification activity of oral commensals like *Prevotella* and *Veillonella* (Doel et al., 2004; Hyde et al., 2014; Sanchez et al., 2014; Hezel and Weitzberg, 2015; Hohensinn et al., 2016; Qu et al., 2016). Due to the significant health consequences that result from RNS activity on oral pathogens, we sought to understand the metabolic alterations that an H_2_O_2_-producing commensal and nitrite have on the oral polymicrobial metabolome, using the notable *S. mutans*-*C. albicans* synergistic interaction as a disease model. In this study, we report that *S. parasanguinis* and nitrite mediate large shifts in the oral metabolome that favor commensal dominance and hinder pathogen survival. Additionally, we show that *S. parasanguinis* conferred a growth advantage on nitrite and the nitrite-trapping antioxidant, thioproline. However, thioproline not only inhibited the growth of the major cariogenic pathogen, *S. mutans*, it impaired the ability of GTFs to synthesize glucan, a critical component of the biofilm matrix that is also utilized by *C. albicans* to synergize with *S. mutans.* Lastly, we show that in the presence of nitrite, *S. parasanguinis* generated metabolites that provide protection against nitrosative stress, including ROS and RNS scavengers. Overall, our findings illustrate that the global antimicrobial activity of *S. parasanguinis* and nitrite drives large shifts in the metabolic signature of cross-kingdom polymicrobial communities and supports the growth and protection of commensal bacteria, whose main function is to maintain homeostasis while restricting the growth of pathogens.

Dietary, bacterial, and host-derived metabolites are important drivers of microbial ecology and antagonism in the oral cavity. For example, carbohydrate metabolites like sucrose support *S. mutans* abundance in oral biofilms, and thus mediate microbial dysbiosis and caries development (Du et al., 2020). In contrast, our study shows that nitrite, a metabolite produced by denitrifying commensals from dietary nitrate (Qu et al., 2016), negates the effects of sucrose and promotes homeostasis since *S. mutans* was inhibited by nitrite and *S. parasanguinis* in sucrose-dependent biofilms. Elevated levels of nitrite and nitrate have been linked to health and homeostasis in the oral cavity (Doel et al., 2004; Hohensinn et al., 2016; Scoffield et al., 2019). Our data demonstrate that nitrite may modulate health by shifting the oral microbial community profile from pathogen to commensal dominance, presumably by generating RNS and metabolites from nitrite metabolism that have a direct impact on pathogen survival. *S. mutans* growth was sensitive to small concentrations of the nitrite-trapping antioxidant thioproline and it directly interfered with the activity of GTFs to synthesize glucan, a major virulence factor and component of the biofilm matrix. Conversely, thioproline promoted the growth rate of *S. parasanguinis.* Further, *S. parasanguinis* cultures grown in nitrite had increased levels of PABA, a ROS scavenger (Hu M et al., 1995). PABA, also produced by the oral commensal *Streptococcus gordonii*, has been shown to promote community development with the periopathogen *Porphyromonas gingivalis*, but extraordinarily can reduce virulence *in vivo* and exopolysaccharide production by *P. gingivalis* (Kuboniwa et al., 2017). Similarly, PABA potentially plays a role in structuring the oral microbiota in the presence of nitrite while restricting bacterial virulence factors, such as glucan. Overall, our data demonstrates that *S. parasanguinis* can utilize nitrite to shift the composition of the oral biofilm from a diseased to healthy state, which has significant health implications.

Oral microbes are constantly exposed to fluxes of nitrogenous intermediates including, nitrate, nitrite, and nitric oxide (Qu et al., 2016), which are all sources of nitrosative stress. In our study, we show that the commensal *S. parasanguinis* has a remarkable ability to tolerate high nitrosative stress or RNS, whereas *S. mutans* and *C. albicans* are more susceptible to RNS. Mechanisms that mediate RNS resistance in *S. parasanguinis* have never been described and generally, how the oral microbiota cope with RNS is poorly understood. Much of what is known about mechanisms used by oral bacteria to resist RNS has been described in *P. gingivalis*. Hydroxylamine reductase and nitric oxide reductase have been shown to be required for *P. gingivalis* tolerance to nitric oxide and growth on nitrite (Boutrin et al., 2012; Lewis et al., 2012; Belvin et al., 2019). However, no known hydroxylamine reductases or nitric oxide reductases have been identified in *S. parasanguinis*. Instead, our findings show that *S. parasanguinis* undergoes global metabolic shifts in RNS scavengers and increases nucleotide metabolism during nitrosative stress conditions. In our study, aromatic compounds from the Shikimate pathway, particularly 3-dehydroshikimate, were significantly increased in *S. parasanguinis* cultures that contained nitrite. Many aromatic compounds have inhibitory activity against nitrosative stress due to their ability to scavenge NO_x_ (Sirimulla et al., 2012; Anam et al., 2020). Moreover, 5-enolpyruvylshikimate-3-phosphate synthase, an enzyme in the Shikimate pathway that participates in the synthesis of aromatic amino acids, is critical for growth on nitrate in *P. aeruginosa* (Filiatrault et al., 2006). Hence, the Shikimate pathway may be critical for nitrogen metabolism and survival during RNS exposure by *S. parasanguinis*. Further, *S. parasanguinis* increased turnover of several nucleosides, predominantly guanosine, inosine, and xanthosine. *Salmonella* undergoes similar reprogramming in nucleotide metabolism in response to nitrosative stress (Fitzsimmons LF et al., 2018). We have previously reported that *S. parasanguinis* generates peroxynitrite in the presence of nitrite (Scoffield and Wu, 2015), a RNS that damages lipids, proteins and DNA (Pacher et al., 2007). *S. parasanguinis* likely induces rapid turnover of nucleotides as an adaptation mechanism for cell survival during RNS exposure to support cell growth, and for reasons that are still unknown, other oral pathogens like *S. mutans* are less capable of generating such a protective response to RNS.

In summary, we propose that a critical function of host and bacterial-derived metabolites is to regulate health and homeostasis by modulating bacterial competition. Oral microbes are routinely exposed to fluxes of nitrogenous intermediates through bacterial denitrification by commensal bacteria like *Prevotella* and *Veillonella* or through the production of other RNS like peroxynitrite from the activity of H_2_O_2_-producing commensal streptococci (Hyde et al., 2014; Scoffield et al., 2019). These varying sources of RNS promote the prevalence of commensal bacteria, and restrict the growth of pathogens and synthesis of virulence factors, which ultimately shapes the biogeography of the oral microbiota. Nevertheless, more studies are needed to understand the molecular mechanisms that protect oral commensal microbes from RNS, but render oral pathogens susceptible. Overall, the modulation of nitrite concentrations in the oral cavity could potentially serve as an effective therapeutic strategy to safeguard oral health.

## Data Availability Statement

The original contributions presented in the study are included in the article/**Supplementary Material**. Further inquiries can be directed to the corresponding author.

## Author Contributions

JTH and JAS were involved in the conception of this study. All authors performed experiments, wrote and edited the manuscript, and approved the manuscript.

## Funding

This project was supported by the following funds and grants awarded to JAS: the University of Alabama at Birmingham Department of Microbiology startup funds, National Institutes of Health/National Institute of Dental and Craniofacial Research Grant R00DE025913, National Institutes of Health/National Institute of General Medical Sciences R35GM142748, and the American Association for Dental Research/Proctor and Gamble Underrepresented Faculty Research Fellowship. JJB is supported by a National Institutes of Health/National Institute of Dental and Craniofacial Research Dental Academic Research Training grant T90DE022736. SNS was supported by the Alabama Louis Stokes for Minority for Participation fellowship funded by the National Science Foundation (1806130), the National Heart, Lung, and Blood Institute (NHLBI) T32 UAB pre-doctoral training program in lung diseases (T32HL134640-03), and is currently funded by a NHLBI NRSA Fellowship (F31HL162487-01).

## Conflict of Interest

The authors declare that the research was conducted in the absence of any commercial or financial relationships that could be construed as a potential conflict of interest.

## Publisher’s Note

All claims expressed in this article are solely those of the authors and do not necessarily represent those of their affiliated organizations, or those of the publisher, the editors and the reviewers. Any product that may be evaluated in this article, or claim that may be made by its manufacturer, is not guaranteed or endorsed by the publisher.
