# Supplementary material for: Nitrite Triggers Reprogramming of the Oral Polymicrobial Metabolome by a Commensal Streptococcus
Source: Front Cell Infect Microbiol. 2022 Mar 1;12:833339. doi: 10.3389/fcimb.2022.833339 (PMC8923425; doi:10.3389/fcimb.2022.833339)
Supplement: Supplementary file 1 [file Table_1.docx]

**Supplemental**

Aa Sp

Ef Sp

**
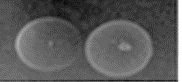
** **
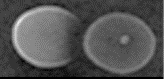
**

control

**
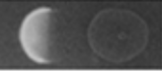
** **
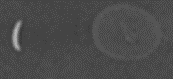
**

2 mM NO_2_

**Figure S1. *S. parasanguinis* and nitrite inhibit endodontic and periodontal pathogens.** Competition assay between *S. parasanguinis* (Sp) and the endodontic pathogen *Enterococcus facaelis* (Ef) (clinical strain) and periodontal pathogen *Aggregatibacter actinomycetemcomitans* VT1169 (Aa). Sp was spotted on Todd Hewitt agar (+/- nitrite) 24 hours before plating Ef or Aa. Plates were incubated for 16 hours at 37º C with 5% CO_2_.


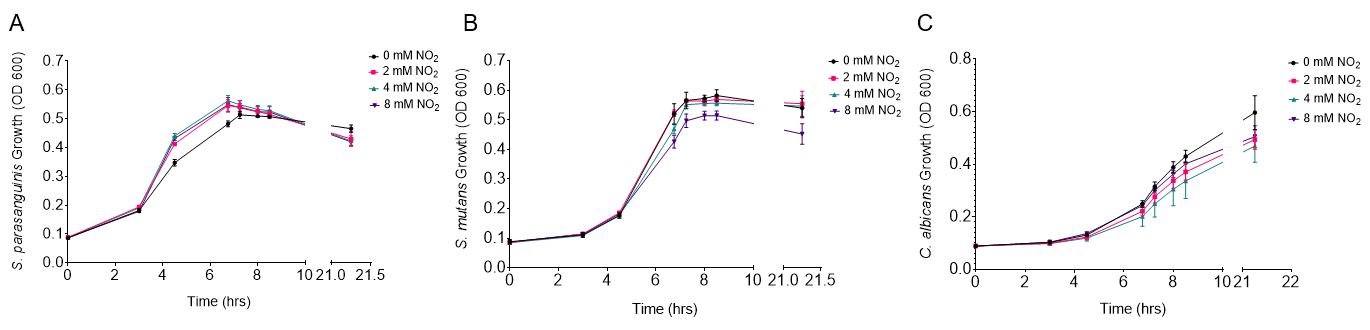
**Figure S2. *S. parasanguinis* displays high tolerance to nitrite.** Bacterial growth curves of (A). *S. parasanguinis*, (B). *S. mutans*, and (C). *C. albicans* grown in TSBYE containing 0, 2, 4, or 8 mM NO_2_. All growth curve cultures were grown in tryptic soy broth containing 0.5% yeast extract at 37 °C with 5% CO_2_. Data are representative of three biological replicates.
